# Improving the autotransporter‐based surface display of enzymes in *Pseudomonas putida* KT2440

**DOI:** 10.1111/1751-7915.13419

**Published:** 2019-05-02

**Authors:** Iasson E. P. Tozakidis, Lena M. Lüken, Alina Üffing, Annika Meyers, Joachim Jose

**Affiliations:** ^1^ Institut für Pharmazeutische und Medizinische Chemie PharmaCampus Westfälische Wilhelms‐Universität Münster Corrensstr. 48 48149 Münster Germany

## Abstract

*Pseudomonas putida* can be used as a host for the autotransporter‐mediated surface display of enzymes (autodisplay), resulting in whole‐cell biocatalysts with recombinant functionalities on their cell envelope. The efficiency of autotransporter‐mediated secretion depends on the N‐terminal signal peptide as well as on the C‐terminal translocator domain of autotransporter fusion proteins. We set out to optimize autodisplay for *P. putida* as the host bacterium by comparing different signal peptides and translocator domains for the surface display of an esterase. The translocator domain did not have a considerable effect on the activity of the whole‐cell catalysts. In contrast, by using the signal peptide of the *P. putida* outer membrane protein OprF, the activity was more than 12‐fold enhanced to 638 mU ml^−1^ OD ^−1^ compared with the signal peptide of *V. cholerae* CtxB (52 mU ml^−1^ OD ^−1^). This positive effect was confirmed with a β‐glucosidase as a second example enzyme. Here, cells expressing the protein with N‐terminal OprF signal peptide showed more than fourfold higher β‐glucosidase activity (181 mU ml^−1^ OD ^−1^) than with the CtxB signal peptide (42 mU ml^−1^ OD ^−1^). SDS‐PAGE and flow cytometry analyses indicated that the increased activities correlated with an increased amount of recombinant protein in the outer membrane and a higher number of enzymes detectable on the cell surface.

## Graphical abstract

In order to improve the autotransporter‐mediated surface display of enzymes in *Pseudomonas putida*, we evaluated different autotransporter translocator domains and signal peptides in terms of their effect on surface display. While the exchange of the translocator domain did not have a significant effect, the utilization of the native OprF signal peptide from *P. putida* resulted in a more than 12‐fold increase of the whole cell catalysts' activities.

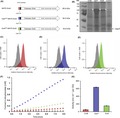

## Introduction

In the last years*, Pseudomonas putida* has been intensively studied with regard to its biotechnological application. This is due to some of the bacterium's beneficial properties, such as metabolic versatility, environmental stress resistance and low cultivation demands, making it particularly valuable for industrial biocatalysis (Poblete‐Castro *et al*., 2012). The best characterized strain is *P. putida* KT2440 (Belda *et al*., 2016), which has been developed towards industrial applications and for which sufficient tools for heterologous gene expression and genetic modifications are available (Lieder *et al*., 2015; Loeschcke and Thies, 2015).

For whole‐cell biocatalytic approaches, it is under certain conditions expedient not to express the desired enzyme intracellularly or to secrete, but instead to display it on the surface of the bacterium, i.e. to transport the enzyme to the outer membrane, rendering it accessible from the extracellular space while maintaining a stable linkage to the cell. This is specifically the method of choice when non‐cell permeable substrates or products come into play or when enzymes require a membrane surrounding (Schuurmann *et al*., 2014). Surface display in Gram‐negative bacteria can be realized by means of the monomeric autotransporter (type Va) secretion pathway (Henderson *et al*., 2004). The utilization of autotransporters for the display of recombinant enzymes (or other proteins) is termed autodisplay (Jose, 2006; Jose and Meyer, 2007; Jose *et al*., 2012).

In earlier studies, we established *Pseudomonas putida* as a host for autodisplay of cellulases and hemicellulases in the context of whole‐cell catalytic biomass degradation (Tozakidis *et al*., 2016; Schulte *et al*., 2017). For this purpose, maximized autotransporter expression (MATE)‐plasmids were used, which encode for fusion proteins consisting of the N‐terminal CtxB signal peptide (SP) from *Vibrio cholerae*, followed by the passenger (that is the enzyme to be displayed) and the translocator domain of the EhaA autotransporter from *E. coli*. The translocator domain can be divided into β1‐domain, α‐helix (together termed linker) and β‐barrel domain. Besides *E. coli* and *P. putida*, MATE plasmids were shown to also be compatible for autodisplay in the ethanologenic bacteria *Zymomonas mobilis* and *Zymobacter palmae* and our experience with several other hosts indicates universal applicability of MATE (Tozakidis *et al*., 2014; Sichwart *et al*., 2015). However, although the term autotransporter suggests an autonomous secretion, it is meanwhile known that several cellular entities participate in the folding of the protein into the outer membrane and that they include species‐specific interactions (Robert *et al*., 2006). It was also found that expression and display levels of heterologous autotransporters correlate with the phylogenetic distance between host and donor species (Marin *et al*., 2010). A previous study has also identified the linker region as an important influence factor on autodisplay (Quehl *et al*., 2017). Besides this, it is obvious that the N‐terminal SP also determines the amount of displayed protein as it mediates its translocation into the periplasm. It therefore seems promising to evaluate different homologues of these domains in order to optimize autodisplay.

Following this idea, we compared different translocator domains, namely EhaA from *E. coli* (Sichwart *et al*., 2015) EstP from *P. putida* (Asler *et al*., 2010) and a combination of EstP α‐helix and EhaA β‐barrel, in terms of their influence on the surface display of an esterase as an example enzyme. This influence, however, turned out to be negligible. Moreover, different signal peptides were tested, i.e. the *V. cholerae* CtxB SP (Maurer *et al*., 1997), the *P. aeruginosa* EstA SP (Wilhelm *et al*., 2007) as well as the native *P. putida* OprF SP (Kragelund *et al*., 1996). The last one had a strong positive effect on the surface display of an esterase as well as a β‐glucosidase with 12.4‐ and 4.3‐fold increases in activity respectively.

## Results and discussion

### Surface display of esterase with different translocator domains

**Figure 1:**
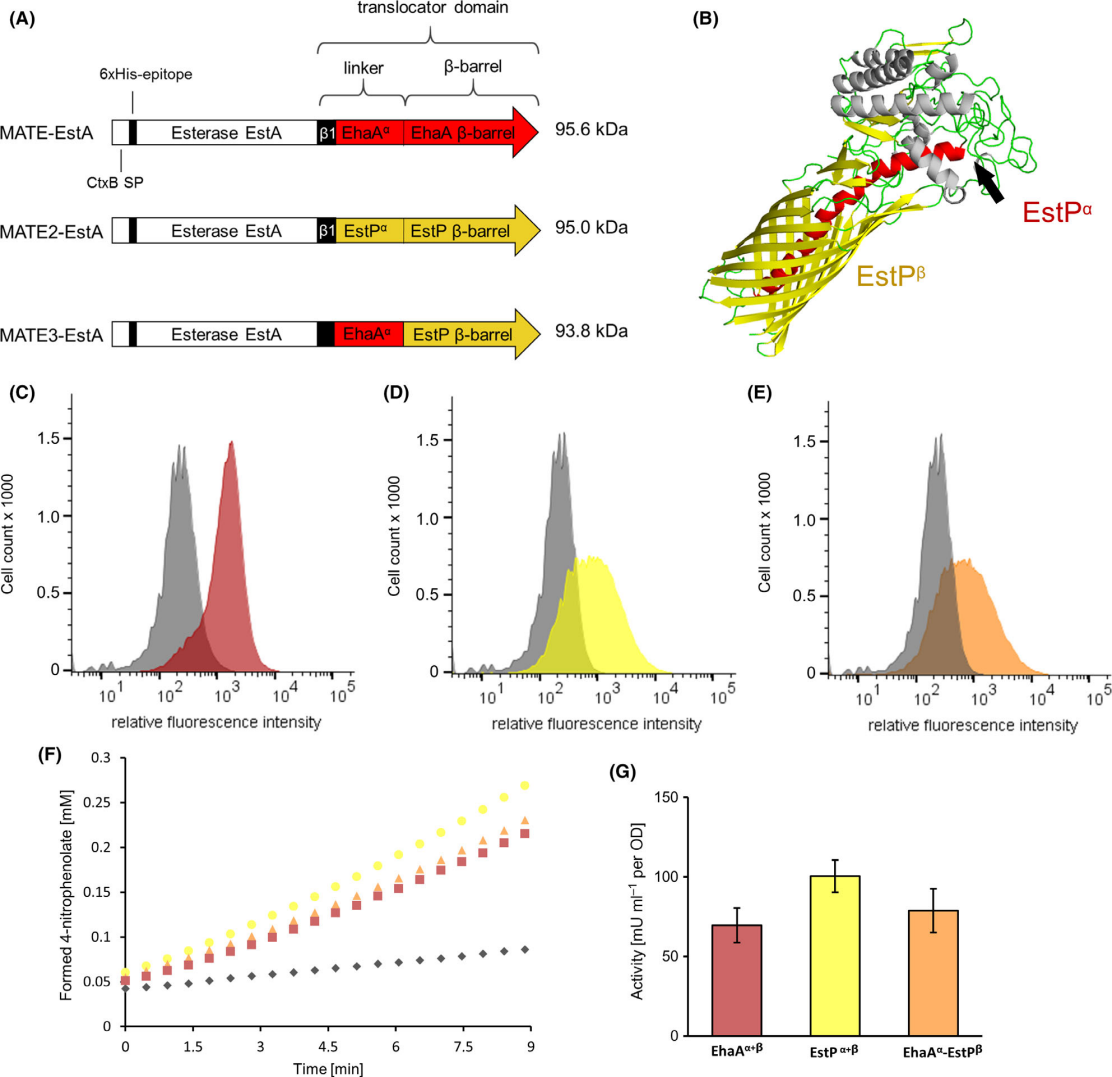
Effect of different translocator domains on the surface display of the esterase EstA.A. Domain structures of MATE‐EstA, MATE2‐EstA and MATE3‐EstA. The calculated molecular weight of each fusion protein is provided in kDa. The respective α‐helix and β‐barrel sequences are provided in Fig. S1.B. Modelled structure of EstP from *P. putida*, revealing its α‐helix (red) and β‐barrel (yellow). The arrow indicates the end of the protein sequence that was used in this study.C. Flow cytometric histograms of *P. putida* pMATE‐EstA, (D) pMATE2‐EstA and (E) pMATE3‐EstA. Cells were labelled with mouse anti‐6xHis and secondary DyLight 633‐conjugated antibodies. Histograms of control cells cultivated without induction of protein expression are shown in grey. Representative histograms from single experiments are shown. Additional control experiments are shown in figure S3.F. Time‐course of 4‐nitrophenolate formation in whole‐cell esterase activity assay and (G) resulting activities performed with *P. putida* pMATE‐EstA (red), pMATE2‐EstA (orange), pMATE3‐EstA (yellow) and control cells without induction of protein expression (grey).

To investigate the influence of different translocator domains on autodisplay of enzymes in *P. putida*, plasmids were constructed that encode for autotransporter fusion proteins consisting (from N‐ to C‐terminus) of the *V. cholerae* CtxB SP, the esterase EstA from *B. gladioli* as an example passenger (Schultheiss *et al*., 2002) with a 6xHis epitope at its N‐terminus and three different translocator domains (Fig. 1A): (i) the translocator domain of the EhaA autotransporter from *E. coli* as described previously (Sichwart *et al*., 2015) and utilized so far for surface display in *P. putida* (Tozakidis *et al*., 2016; Schulte *et al*., 2017). This protein (and the encoding plasmid) was named MATE‐EstA according to the previous terminology. (ii) A translocator in which the α‐helix and β‐barrel of EhaA were replaced by the corresponding domains from the EstP autotransporter from *P. putida* KT2440 (Asler *et al*., 2010). To define these domains, the tertiary structure of EstP was modelled via RaptorX (Kallberg *et al*., 2012), and the α‐helix and β‐barrel relevant for autotransporter secretion could be identified (Figure 1B). The resulting protein was termed MATE2‐EstA. (iii) A translocator that combines the α‐helix of EhaA and the β‐barrel domain of EstP (termed MATE3‐EstA). The β1‐domain derived from *E. coli* EhaA remained constant in all constructs.

The strains as obtained after transformation, *P. putida* pMATE‐EstA, pMATE2‐EstA and pMATE3‐EstA, were cultivated and the encoded fusion proteins were expressed under the control of the araBAD promotor. Strains without induction of protein expression were used as controls. To determine the expression of surface displayed esterase, the cultures were harvested and washed, and the cells were incubated with anti‐6xHis antibodies. After washing to get rid of unbound antibodies, cells were treated with secondary DyLight 633‐coupled antibodies, washed again and subjected to flow cytometer analysis. As the antibodies are too large to penetrate the membrane, they can only interact with their epitope in case it is accessible from the outside. Fluorescence intensity of the cells should therefore be proportional to the number of enzymes displayed on their surface. As seen in Fig. 1C–E, all strains expressing one of the autotransporter fusion proteins showed enhanced fluorescence compared to the control, with similar mean fluorescence (mf) values (MATE‐EstA: 1591; MATE2‐EstA: 1307; MATE3‐EstA: 1217), indicating that they contained correctly folded autotransporters and displayed EstA on their surface.

To evaluate the influence of the different translocator domains on the activity of *P. putida* whole‐cell catalysts, the strains were cultivated, protein expression was induced, and cell suspensions with an optical density at 578 nm (OD) of 0.2 were incubated with 4‐nitrophenyl acetate at a temperature of 30°C to photometrically monitor the formation of the reaction product 4‐nitrophenolate (Fig. 1F and G). Surface display of the esterase by means of MATE2‐EstA resulted in 44% higher activity (101 mU ml^−1^ OD^−1^) compared with MATE‐EstA (70 mU ml^−1^ OD^−1^). The fusion of EhaA α‐helix and EstP β‐barrel (MATE3‐EstA) had no significant positive effect on the activity (79 mU ml^−1^ OD^−1^).

Considering these findings, it is either conceivable that the compatibility of the EhaA autotransporter with *P. putida* periplasmic chaperones is such high that the secretion cannot be optimized significantly at this point or that the recognition of the translocator domain is not the limiting step of the secretion mechanism. In the latter case, the transport of the autotransporter fusion protein into the periplasm and consequently the recognition of the N‐terminal signal peptide should represent the bottleneck of surface display. We therefore shifted our focus on substituting the signal peptide in order to optimize autodisplay for *P. putida*.

### Surface display of esterase with different signal peptides

**Figure 2:**
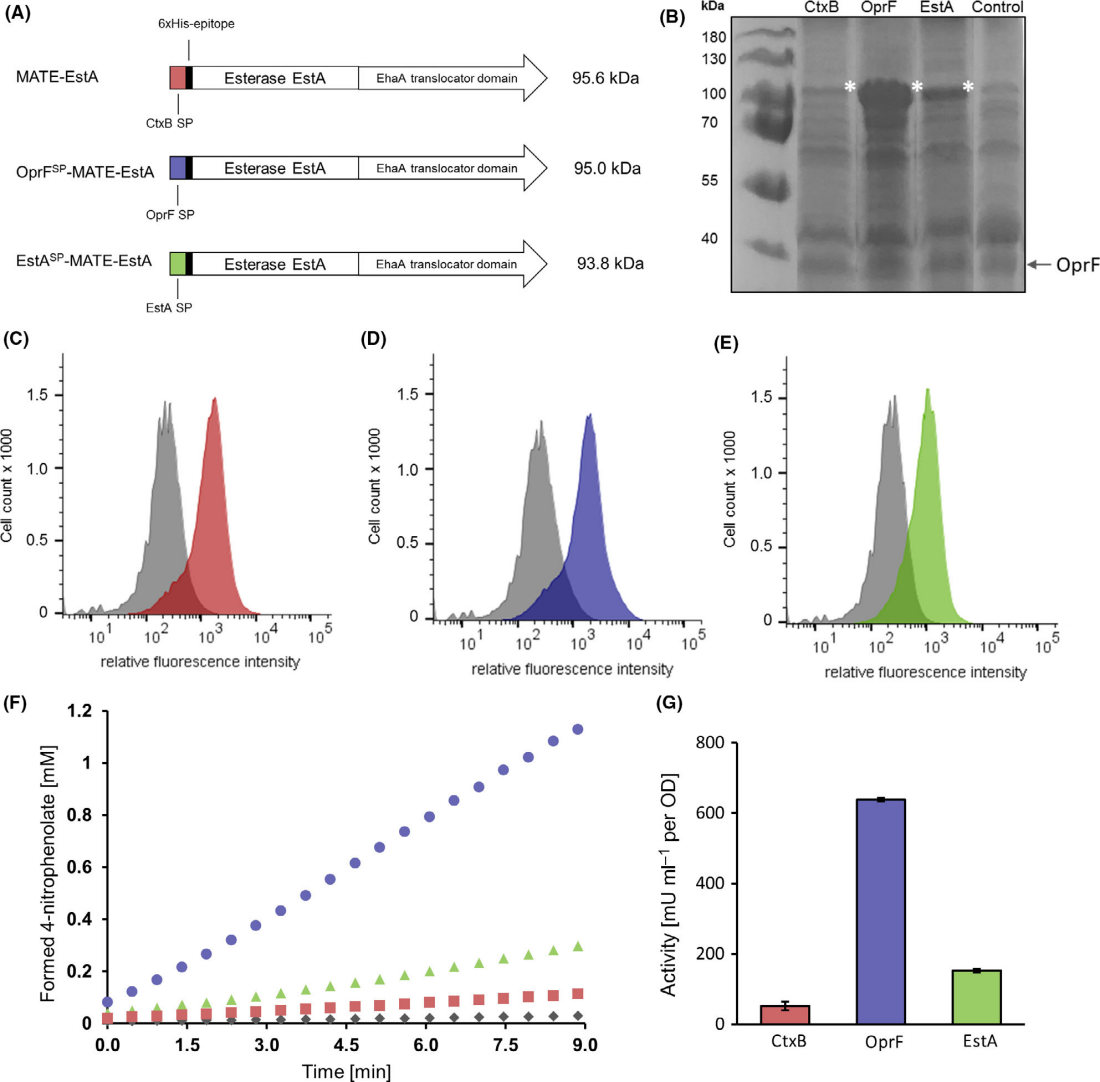
Effect of different signal peptides on the surface display of the esterase EstA.A. Domain structures of MATE‐EstA, OprF^SP^‐MATE‐EstA and EstA^SP^‐MATE‐EstA. The calculated molecular weight of each fusion protein is provided in kDa.B. SDS‐PAGE of outer membrane proteins of *P. putida* expressing autotransporter fusion proteins with different signal peptides. An arrow indicates the protein band assigned to the outer membrane protein OprF used as loading control. *P. putida* with an empty vector was used as control. The asterisks depict the protein bands assigned to the recombinant fusion proteins.C. Flow cytometric histograms of *P. putida* pMATE‐EstA, (D) pOprF^SP^‐MATE‐EstA and (E) pEstA^SP^‐MATE‐EstA. Cells were labelled with mouse anti‐6xHis and secondary DyLight 633‐conjugated antibodies. Histograms of control cells cultivated without induction of protein expression are shown in grey. Representative histograms from single experiments are presented. Additional control experiments are shown in figure S3.F. Time‐course of 4‐nitrophenolate formation in whole‐cell esterase activity assay and (G) resulting activities of *P. putida* pMATE‐EstA (red), pOprF^SP^‐MATE‐EstA (blue), pEstA^SP^‐MATE‐EstA (green) and control cells without induction of protein expression (grey). Standard deviations of biological triplicates are indicated.

Plasmids were constructed that encode for autotransporter fusion proteins with different signal peptides: (i) the routinely used CtxB SP from *V. cholerae* (Jose and Meyer, 2007), i.e. the protein MATE‐EstA; (ii) the SP of the outer membrane protein OprF from *P. putida* (Kragelund *et al*., 1996), resulting in OprF^SP^‐MATE‐EstA; OprF is a dominant outer membrane protein of *P. putida* KT2440 that plays a similar role as OmpA in *E. coli* (Wang, 2002); and (iii) the SP of EstA from *Pseudomonas aeruginosa*, resulting in the protein EstA^SP^‐MATE‐EstA. In *P. aeruginosa*, this SP is involved in the surface display of a virulence factor (Wilhelm *et al*., 2007). The following C‐terminal parts of the fusion proteins remained as described before, including the EhaA translocator. The corresponding domain structures are shown in Fig. 2A, the amino acid sequences of the signal peptides are depicted in Fig. S2.

After transforming *P. putida* with these plasmids, cultivating the resulting strains and inducing protein expression, outer membrane proteins were isolated and separated *via* SDS‐PAGE (Fig. 2B). In all samples except the control (*P. putida* with empty vector), a protein band at an apparent molecular weight of approximately 100 kDa was visible. This band was assigned to the recombinant autotransporter fusion proteins with calculated molecular weights between 91 and 93 kDa after release of the SP in all of the three samples. This protein band was most pronounced in samples of *P. putida* pOprF^SP^‐MATE‐EstA, whereas the faintest band appeared in samples of *P. putida* pMATE‐EstA, giving a first hint on the different translocation efficiencies of the signal peptides. Alongside the CtxB SP, both the OprF SP and the EstA SP were therefore functional in terms of translocating the protein into the periplasm.

Next, the strains were analysed *via* flow cytometry as described above (Fig. 2C–E). All three of them exhibited enhanced fluorescence intensities compared with the controls, indicating the surface localization of EstA. Consistently with the observed protein band intensities in the SDS‐PAGE, *P. putida* pOprF^SP^‐MATE‐EstA showed the highest mf of 2064. Contrary to the faint band in the SDS‐PAGE, *P. putida* pMATE‐EstA had a higher mf (1591) than *P. putida* pEstA^SP^‐MATE‐EstA (1035). At this point, it is unclear whether the secretion process itself, the subsequent labelling with antibodies or both factors are responsible for the observed difference between protein amount in the outer membrane and detectable EstA on the cell surface.

Whole‐cell esterase activity tests of each strain were performed as described above and revealed that replacing the CtxB SP had much stronger influence on the activity than replacing the EhaA translocator (Fig. 2F and G). Cells expressing the fusion protein with the OprF SP possessed a 12.4‐fold higher esterase activity (638 mU ml OD^−1^) than cells with the CtxB SP (52 mU ml OD^−1^). The activity of cells with the EstA SP was between these values with 152 mU ml^−1^ OD^−1^.

To assess whether these effects originated from a codon usage bias, the codon adaption index (CAI) of the CtxB‐ and the OprF‐sequences were calculated using the online tool JCat (Grote *et al*., 2005). The CAI value of the CtxB sequence was significantly higher (0.54) than that of the OprF sequence (0.35), indicating that the higher activities of *P. putida* pOprF^SP^‐MATE‐EstA cells were not an effect of codon bias. Additionally, RBS calculator (Salis, 2011) was used to predict the translation rates of the described constructs, which can be influenced by secondary structures of the gene transcripts. The translation rate for pMATE‐EstA was more than twice as high as for pOprF^SP^‐MATE‐EstA (16 542 vs. 7628). Hence, this parameter could also not account for the observed differences in whole‐cell activities. It can thus be assumed that the signal peptides have different secretion efficiencies in *P. putida*.

It was observed that all strains expressing the autotransporter fusion proteins exhibited an increased sensitivity towards antibiotics, which could be an effect of the increased number of β‐barrel proteins within the outer membrane (Fig. S4). Also, the viability of the strains decreased significantly by a factor of 1000 in terms of colony‐forming units. This could reflect the burden of protein expression or an impairment of the cell's outer membranes by the β‐barrel proteins (Fig. S5).

### Surface display of β‐glucosidase with different signal peptides

**Figure 3:**
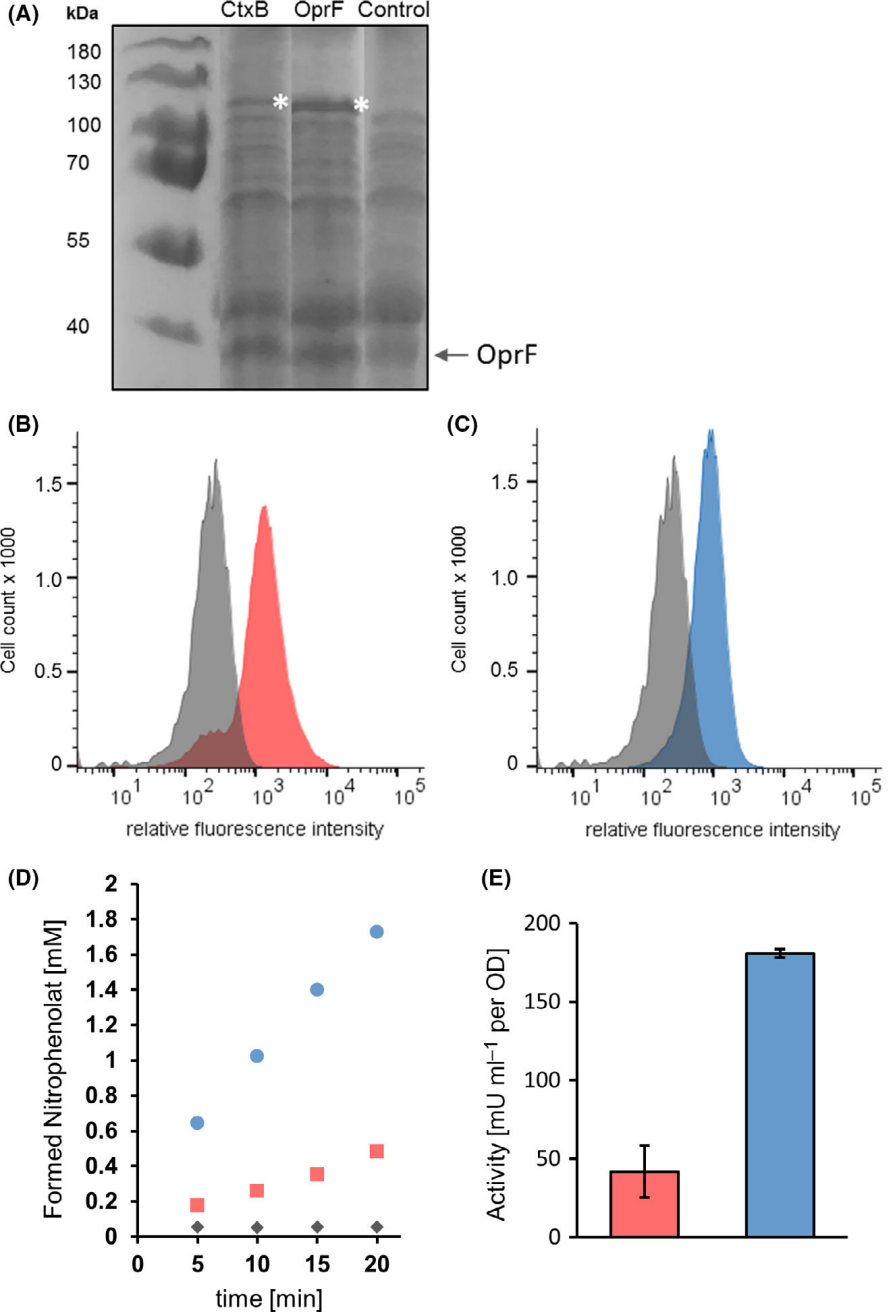
Effect of different signal peptides on the surface display of the β‐glucosidase BglA.A. SDS‐PAGE of outer membrane isolates. An arrow indicates the protein band assigned to the outer membrane protein OprF used as loading control. *P. putida* with an empty vector was used as control. The asterisks depict the protein bands assigned to the recombinant fusion proteins.B. Flow cytometric histograms of *P. putida* pMATE‐BglA and (C) pOprF^SP^‐MATE‐BglA. Cells were labelled with mouse anti‐6xHis and secondary DyLight 633‐conjugated antibodies. Histograms of control cells cultivated without induction of protein expression are shown in grey. Representative histograms from single experiments are presented.D. Time‐course of 4‐nitrophenolate formation in whole‐cell β‐glucosidase activity assay and (E) resulting activities of *P. putida* pMATE‐BglA (red), pOprF^SP^‐MATE‐BglA (blue) and control cells without plasmid (grey). Standard deviations of biological triplicates are indicated.

In order to confirm the beneficial effect of the OprF SP on the surface expression with autodisplay, as found with esterase, we investigated a second passenger protein, the β‐glucosidase BglA from *Caldicellulosiruptor saccharolyticus* (Hong *et al*., 2009). To this end, the plasmids pMATE‐BglA and pOprF^SP^‐MATE‐BglA were constructed, encoding for the autotransporter fusion proteins as described before, with either CtxB or OprF SP at the N‐terminus, followed by BglA as the passenger domain and the EhaA translocator. As before, the proteins included a 6xHis epitope for immunological detection. After cultivation of the strains and induction of protein expression, outer membrane proteins were isolated and separated by SDS‐PAGE (Fig. 3A). A protein band at an apparent molecular weight between 100 and 130 kDa was detected in samples of *P. putida* pOprF^SP^‐MATE‐BglA and, to a much lesser extent, in samples of *P. putida* pMATE‐BglA. It could be assigned to the recombinant fusion proteins with a calculated theoretical molecular mass between 111 and 112 kDa after release of the SP during transport across the inner membrane. With both signal peptides, surface display could be demonstrated in flow cytometry experiments (Fig. 3B and C). In addition, β‐glucosidase activity of these cells using 4‐nitrophenyl‐β‐D‐glucopyranoside as substrate was 4.3‐fold higher (181 mU ml^−1 ^OD^−1^) than the activity of cells with CtxB SP (42 mU ml^−1^ OD^−1^) (Fig. 3D and E). Hence, replacing the CtxB SP by the OprF SP had a similar impact on autodisplay of the β‐glucosidase as on autodisplay of the esterase, although the effect seems to be biased by the passenger domain.

## Conclusion

These findings support the hypothesis that the transport of fusion proteins across the inner membrane of the bacterium could represent a bottleneck when using MATE for autodisplay in *P. putida* (Tozakidis *et al*., 2016; Schulte *et al*., 2017). Transport efficiency appears severely affected by the SP, but not by the translocator domains used. This study helps optimizing *P. putida* whole‐cell biocatalysts with surface displayed enzymes and identified the OprF signal peptide as well suited for autodisplay in this host.

## Experimental procedures

### Bacterial strains and culture conditions

Cloning was carried out in *Escherichia coli* DH5α (DSM No.: 6897). The cells were cultivated either in lysogeny broth (LB) medium at 37°C and 200 rpm or on LB agar plates at 37°C, both containing 50 μg ml^−1^ kanamycin. *P. putida* KT2440 (DSM No.: 6125) was cultivated either in LB medium or on LB agar plates, with 50 μg ml^−1^ kanamycin when necessary, at 30°C. Main cultures were inoculated to OD of 0.05 from an overnight culture and cultivated in shaking flasks at 30°C and 200 rpm. At OD values of 0.5, gene expression was induced by the addition of 0.2% of L‐(+)‐Arabinose for 2 h at 30°C and 200 rpm.

### Construction of plasmids

All constructed plasmids are based on pMATE‐EstA (Tozakidis *et al*., 2014). For the construction of pMATE2‐EstA, the sequence encoding for the α‐helix and β‐barrel of EstP (Asler *et al*., 2010) was amplified from the chromosomal DNA of *P. putida* KT2440. The sequence was inserted into pMATE‐EstA instead of the EhaA α‐helix and β‐barrel *via* In‐Fusion^®^ Cloning as described by the manufacturer. For the construction of pMATE3‐EstA, the sequence encoding for the β‐barrel of EstP was amplified and inserted into pMATE‐EstA instead of the EhaA β‐barrel. For the construction of pOprF^SP^‐MATE‐EstA, the DNA sequence of the signal peptide (SP) of OprF (Kragelund *et al*., 1996) was amplified from the chromosomal DNA of *P. putida* KT2440. The sequence of the original CtxB SP of MATE‐EstA was substituted by the OprF SP *via* In‐Fusion^®^ Cloning. For the construction of pEstA^SP^‐MATE‐EstA, the CtxB SP sequence was substituted by the SP of *P. aeruginosa* EstA (Wilhelm *et al*., 2007). The DNA sequence of the EstA SP was synthesized commercially and inserted into MATE‐EstA *via* In‐Fusion^®^ Cloning. For the construction of pMATE‐BglA and pOprF^SP^‐MATE‐BglA, the DNA sequence of *C. saccharolyticus* BglA (NCBI GenBank: P10482) was inserted into the plasmids pMATE‐EstA and pOprF^SP^‐MATE‐EstA *via* restriction/ligation cloning with XhoI and KpnI. To enable the translation of full‐length MATE‐BglA and OprF^SP^‐MATE‐BglA, the stop codon of *C. saccharolyticus* BglA had to be removed beforehand. All cloning primers are listed in Table S1. The sequences of all constructed plasmids were verified by Sanger sequencing (Microsynth Seqlab, Göttingen, Germany).

### Flow cytometry analyses

For flow cytometry analyses, the cells were cultivated in LB medium to an OD of 0.5 at 30°C and 200 rpm. Protein expression was induced by supplementation of 0.2% of L‐(+)‐Arabinose for 2 h. The cells were then harvested and diluted with PBS to an OD of 0.4. After three rounds of centrifugation and washing with particle‐free PBS (12 000 *g*, 1 min), the cells were suspended in 100 μl primary antibody solution (THE^TM^ His tag Antibody, mouse, 1:100 in PBS; GenScript, Piscataway, NJ, USA) and incubated for 45 min at room temperature (RT). The cells were again centrifuged and washed three times with particle‐free PBS (12 000 *g*, 2 min) and suspended in 100 μl of a secondary antiserum in PBS (anti‐mouse IgG (H+L) Secondary Antibody, DyLight 633‐coupled, goat, 1:100 in PBS; Thermo Fisher Scientific, Waltham, MA, USA) and incubated for 60 min at RT in the dark. The cells were centrifuged and washed three times with particle‐free PBS (12 000 *g*, 2 × 2 min, 2 × 1 min) and each pellet was suspended in 500 μl particle‐free PBS. The fluorescence of the cells was analysed with a FACSAria III (Becton Dickinson, Heidelberg, Germany). 50000 events of every sample were analysed. The DyLight 633 fluorescence was excited at 633 nm and analysed at 660/20 nm (band‐pass filter, 537 V).

### Photometric activity assays

For esterase activity determination, the cells were sedimented, washed with 50 mM sodium phosphate buffer pH 7 and suspended in the same buffer to an OD of 1. 40 μl of this suspension was mixed with 160 μl of a 4‐nitrophenyl acetate solution (4 mM in 50 mM sodium phosphate buffer) in a microtiter plate and incubated at 30°C under shaking. At given time points, the absorption at 405 nm was measured with a microtiter plate reader. For the β‐glucosidase activity assay, the cells were washed and suspended in 100 mM sodium citrate buffer pH 6 to an OD of 1, mixed in a 1:1 ratio with a 4‐nitrophenyl‐β‐d‐glucopyranoside solution (5 mM in 100 mM sodium citrate buffer) and incubated at 55°C under shaking. At given time points, the reaction samples were centrifuged, 50 μl of the supernatant was mixed with 50 μl of a 2 M Na_2_CO_3_ solution and absorption was detected at 405 nm. In both assays, the concentration of the generated 4‐nitrophenol was calculated by means of 4‐nitrophenol calibration curves.

### Outer membrane protein isolation and separation

For the isolation of membrane proteins, adapted protocols of Lee *et al*. (2005) and Park *et al*. (2015) were used. 100 ml of cell suspensions were cultivated to an OD of 0.5 and induced with 0.2% of L‐(+)‐Arabinose. Protein expression was induced for 2 h, and the cells were harvested (5000 *g*, 5 min, 4°C). The cell pellets were suspended in 10 ml of Tris–HCl buffer (10 mM, pH 7.5) and centrifuged (5000 *g*, 5 min, 4°C). The pellets were suspended in 5 ml Tris–HCl buffer (10 mM, pH 7.5) with 2 mM PMSF, 10 μg ml^−1^ aprotinin and 4 μg ml^−1^ pepstatin. Crude extracts of *P. putida* cells were prepared by three cycles of sonication (40 s, 25% of maximum output). Each crude extract was supplemented with 0.05 mg ml^−1^ DNase I and incubated for 20 min at 37°C. 2 ml of each crude extract was centrifuged (8000 *g*, 7 min, 4°C) to remove partially disrupted cells. The supernatants were centrifuged (20 000 *g*, 30 min, 4°C) to isolate membrane proteins and lipid layers. The pellets were suspended in Tris‐HCl buffer (10 mM, pH 7.5) and centrifuged again (20 000 *g*, 30 min, 4°C). This step was repeated. The supernatant was then removed, and the membrane proteins suspended in Millipore water. The suspensions were supplemented with SDS sample buffer (containing 30 mg ml^−1^ dithiothreitol), incubated for 30 min at 95°C and separated *via* sodium dodecyl sulphate‐polyacrylamide gel electrophoresis (SDS‐PAGE) with PageRuler Unstained Protein Ladder as size standard. The outer membrane protein OprF with a molecular weight of 35 kDa was used as a loading control (Hancock *et al*., 1990). For the analysis, 12.5% acrylamide gels were used and afterwards stained in ProBlueSafe Stain (GiottoBiotech, Sesto Fiorentino, Italy) and de‐stained with water.

## Conflict of interest

None declared.
